# Linking warm conveyor belts to the TEM circulation in Northern Hemisphere midlatitude winter

**DOI:** 10.1038/s41612-026-01435-3

**Published:** 2026-05-15

**Authors:** Yunsung Hwang, Changhyun Yoo, Sukyoung Lee, Michael Sprenger, Marc Federer

**Affiliations:** 1 Severe Storm Research Center, Ewha Womans University, Seoul, South Korea; 2 Department of Climate and Energy Systems Engineering, Ewha Womans University, Seoul, Republic of Korea; 3 Department of Meteorology and Atmospheric Science, The Pennsylvania State University, University Park, PA USA; 4 Institute for Atmospheric and Climate Science, ETH Zurich, Zurich, Switzerland; 5 Department of Earth, Atmospheric and Planetary Sciences, Massachusetts Institute of Technology, Cambridge, MA USA

**Keywords:** Climate sciences, Environmental sciences

## Abstract

Warm conveyor belts (WCBs) are key ascending airstreams in extratropical cyclones that shape midlatitude moisture transport and precipitation. Here we show that WCB activity is tightly linked to the large-scale transformed Eulerian-mean (TEM) circulation in Northern Hemisphere winter. The climatological WCB frequency aligns with the rising branch of the TEM circulation, and the two exhibit strong day-to-day covariability. Variations in WCB activity correspond systematically to changes in large-scale eddy fluxes: enhanced eddy momentum flux (EMF) shifts WCB activity poleward through changes in ascent rate, while enhanced eddy heat flux (EHF) primarily strengthens WCB activity downstream over the Pacific, with little meridional displacement. WCBs account for up to 89% of EMF-related and 56% of EHF-related precipitation anomalies, with the exact fraction varying by latitude. These results demonstrate that the TEM framework provides insight into the zonal-mean imprint of WCB activity and associated precipitation, and how these relationships may change in the future.

## Introduction

The transformed Eulerian mean (TEM) framework describes the residual mean circulation arising from eddy fluxes and diabatic forcing^1,2^. Unlike the conventional Eulerian mean, the TEM formulation accounts for eddy-driven transport of air parcels, providing a representation of large-scale material motion. However, it remains unclear how this zonal-mean residual circulation relates to the zonally varying material transport that produces clouds and precipitation. This motivates the use of physically identifiable Lagrangian features to bridge the TEM framework and a weather-feature-based perspective. Warm conveyor belts (WCBs) are spatially and temporally coherent Lagrangian airstreams, originating in the warm sector of extratropical cyclones and ascending slantwise to the upper troposphere^3–5^. Through their intense moisture transport and dominant role in midlatitude precipitation, WCBs may collectively shape the diabatic heating that modulates the vertical motion of the TEM circulation and its associated zonal-mean precipitation.

WCBs are a primary source of midlatitude precipitation through large-scale stratiform ascent and embedded convection^5–8^. Previous studies have shown that eddy-driven vertical motion, as represented by the TEM framework, strongly influences the zonal-mean precipitation distribution^9,10^. However, the extent to which this vertical motion and precipitation are dynamically linked to WCB activity remains uncertain. Furthermore, the influence of large-scale eddy fluxes of momentum and heat (EMF and EHF) on WCB frequency, structure, and their associated precipitation has not been quantified.

Yoo and Lee^9^ investigated the relationship between TEM vertical motion and precipitation. They postulated that, based on TEM transport of moisture, the TEM circulation may represent the zonal-mean signature of cyclone-scale WCB processes. However, direct analysis of WCB metrics was not included in their study. Other works have examined how WCBs interact with large-scale circulation features such as jet streams, Rossby waves, and upper-level waveguides^11–15^. These studies show that WCBs can, for example, amplify downstream ridges and influence the development of atmospheric blocks. In particular, it has been shown that the interaction of WCBs with the larger-scale flow varies systematically with their outflow latitude^15^. Yet, this body of work does not directly address how WCB activity contributes to, or is represented within, the zonal-mean circulation described by the TEM framework.

In this study, we examine the links between WCB activity, eddy fluxes, and the TEM circulation to determine how cyclone-scale ascent is reflected in the zonal-mean vertical motion and precipitation. We first show that the climatological WCB activity is closely linked to the TEM residual circulation and its associated vertical motion and precipitation. We then investigate how the frequency, structure, and trajectory characteristics of WCBs relate to variations in the EMF and EHF. Finally, we assess the extent to which changes in WCB activity account for the precipitation response to eddy flux variations, and how these relationships differ between EMF and EHF forcing.

## Results

### TEM circulation–WCB–precipitation relationship

**Fig. 1:**
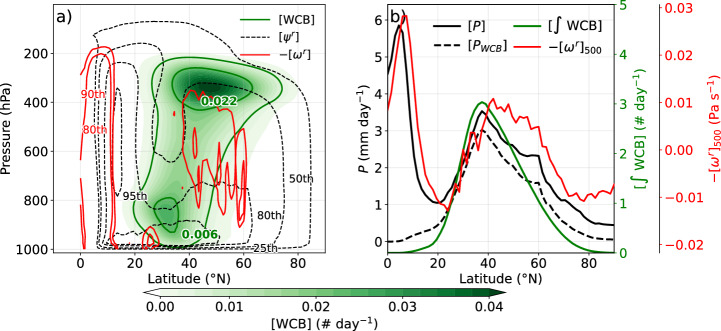
Winter climatology of the TEM circulation, zonal-mean precipitation, and WCB frequency. **a** The TEM streamfunction, calculated from Eq. (1), depicts a clockwise circulation descending branch near 20°N and an ascending branch near 40°N (black dashed contour, 25th: 2.42 × 10^9^, 50th: 1.81 × 10^10^, 80th: 4.69 × 10^10^, and 95th: 6.61 × 10^10^ percentiles are shown in kg s⁻¹). The 90th and 95th percentiles (i.e., 5.21 × 10^−3^ and 1.09 × 10^−2^ Pa s^−1^) of the TEM vertical velocity ($-\left[{\omega }^{r}\right]$) are shown in red contours. Zonal-mean WCB frequency is shown as shading, with its 75th and 95th percentiles (i.e., 0.006 and 0.022 day^−1^; green contours). **b** Zonal-mean precipitation (black line, P), the WCB-related precipitation (P_WCB_, black dashed line), TEM vertical velocity at 500 hPa (red line), and the vertically integrated WCB frequency (green line) are shown.

We begin by examining the climatological relationship between the TEM circulation and the WCB frequency (Fig. 1). The key feature is that the zonal-mean WCB frequency is collocated with the rising branch of the TEM circulation in the midlatitude (Fig. 1a). The TEM streamfunction (black contours; Eq. 1) indicates a clockwise circulation in the latitude–pressure plane, with a U-shaped dip structure located between approximately 20°N and 50°N. Because the TEM vertical velocity is associated with the meridional gradient of the streamfunction (Eq. 2), ascending motion exhibits a pronounced maximum near 40°N in the mid troposphere (red contours). Consistent with this structure, the climatological WCB frequency also reaches a maximum near 40°N (green contours and shading). Moreover, both the WCB frequency and the TEM streamfunction display a distinct south–north tilt with height, highlighting their similar vertical structures. A similar slantwise overturning structure is also evident in the isentropic mass streamfunction^16,17^. The zonal-mean WCB frequency has not been explicitly shown in previous studies, although basin-wide characteristics have been documented (Fig. 8 in Joos et al.^18^), and the zonal-mean structure can also be inferred from longitude–latitude climatologies of inflow, ascent, and outflow. The agreement becomes even clearer when comparing the vertically integrated WCB frequency with the TEM vertical velocity at 500 hPa (green and red lines in Fig. 1b).

Next, we examine how much of the precipitation is linked to WCB activity. Because the zonal-mean precipitation is strongly linked to the TEM vertical motion^9^ (also see black and red lines in Fig. 1b), and the TEM vertical motion corresponds closely to the zonal-mean WCB frequency, it is reasonable to expect that a substantial portion of the total precipitation is associated with WCB activity. Indeed, the zonal-mean precipitation associated with the WCB exhibits large values across the midlatitudes, with a peak centered near 35°N (black dashed line in Fig. 1b). On average, the WCB-related precipitation accounts for about 82% of total precipitation between 30°N and 60°N, underscoring its dominant contribution. This finding itself is not new, as the key role of WCBs in midlatitude precipitation has been well documented^5,6^. Here, we establish this as a basis for the subsequent analysis, which investigates how much of the variability in precipitation associated with the TEM circulation can also be attributed to WCB activity.

**Fig. 2:**
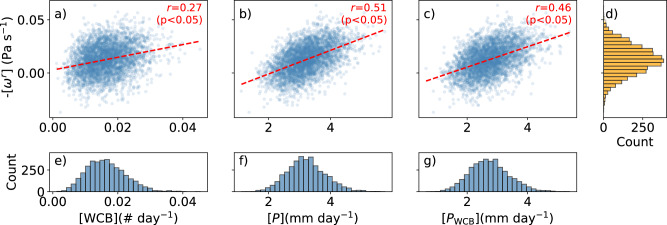
TEM vertical velocity–WCB–precipitation relationships. Scatterplots and frequency distributions illustrating the relationship between the TEM vertical velocity ($-\left[{\omega }^{r}\right]$) and **a** the zonal-mean WCB frequency, both averaged over 650–400 hPa and 35°N–45°N, **b** zonal-mean (35°N–45°N) precipitation, and **c** zonal-mean (35°N–45°N) WCB-related precipitation. Red lines show least-squares linear fits, with correlation coefficients shown. Panel **d** shows the distribution of $-\left[{\omega }^{r}\right]$ averaged over 650–400 hPa and 35°N–45°N. **e**–**g** show the distributions of the zonal-mean WCB frequency, zonal-mean precipitation, and zonal-mean WCB-related precipitation.

This spatial correspondence supports the hypothesis of Yoo and Lee^9^ that the TEM circulation can be interpreted as the zonal-mean representation of the WCB. To further investigate this connection, we examine whether the covariability between the TEM circulation and precipitation is partly mediated by variations in WCB activity. When the WCB frequency is averaged over 650–400 hPa and 35°N–45°N, it shows a weak but significant positive correlation with the reversed TEM vertical velocity $-[{\omega }^{r}]$ (*r* = 0.27; Fig. 2a). Notably, this occurs even though the WCB frequency is intrinsically skewed toward positive values (Fig. 2e), whereas the vertical-velocity distribution is more Gaussian (Fig. 2d). Stronger correlations emerge between the TEM vertical velocity and total precipitation (*r* = 0.51; Fig. 2b, f) and between the TEM vertical velocity and WCB-related precipitation (*r* = 0.46; Fig. 2c, g).

The modest correlation between WCB frequency and TEM vertical velocity may arise because the two diagnostics represent different aspects of the circulation. WCB frequency is an occurrence metric based on identified trajectories and is therefore not a direct measure of ascent strength. In contrast, precipitation is more directly tied to vertical motion and condensation, which helps explain why TEM vertical velocity shows stronger correlations with both total and WCB-related precipitation. Nonetheless, the correlation between WCB frequency and TEM vertical velocity remains statistically significant at the 95% confidence level.

### WCB changes associated with large-scale eddy fluxes

We now turn to the relationship between large-scale eddy fluxes and WCB frequency. Because the TEM residual circulation is forced by EMF and EHF divergences, and because WCBs are embedded within storm-track eddies that contribute to these fluxes, variations in EMF and EHF provide a natural large-scale context for interpreting changes in WCB activity. This analysis first evaluates whether, and to what extent, variations in WCB activity are consistent with changes in the TEM circulation and whether these variations help explain corresponding changes in precipitation.

**Fig. 3:**
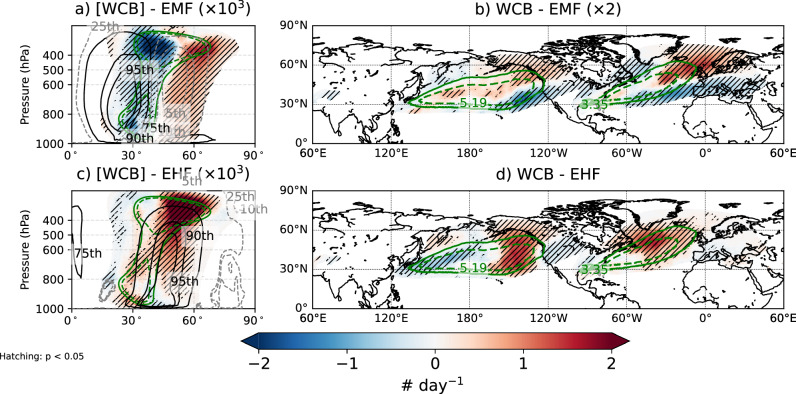
WCB frequency response to EMF and EHF. Regression of daily WCB frequency onto the EMF and EHF indices. Shading denotes regression slopes representing the change in WCB frequency per unit change in **a**, **b** EMF and **c**, **d** EHF, shown in **a**, **c** pressure-latitude and **b**, **d** latitude-longitude coordinates. Values in **a**, **c** are multiplied by 10^3^, and values in **b** by 2. Hatching marks regions where the regression coefficient is significant at the 95% confidence level. Black contours indicate regression of the TEM streamfunction, with positive values in black solid contours (75th: 6.89 × 10^9^, 90th: 2.06 × 10^10^, 95th: 2.68 × 10^10^ percentiles in kg s⁻¹) and negative values in gray dashed contours (5th: −1.08 × 10^8^, 10th: −6.07 × 10^6^, 25th: 8.75 × 10^7^ percentiles in kg s⁻¹). Green contours outline the 90th (solid) and 95th (dashed) percentiles: 0.006 and 0.02 day^−1^ for (**a**, **c**) and 3.36 and 5.20 day^−1^ for **b**, **d** of the climatological WCB frequency.

Regression of the zonal-mean WCB frequency onto the EMF index reveals a meridional dipole pattern, with reduced activity near 35°N and enhanced activity near 50°N (shading in Fig. 3a). These WCB anomalies indicate a poleward shift from their climatological position (green contours) and are consistent with the TEM streamfunction anomalies (black and gray contours), because the TEM vertical velocity is proportional to the meridional gradient of the TEM streamfunction (Eq. 2). The horizontal pattern further supports this relationship, showing a coherent poleward shift in WCB frequency over both ocean basins (Fig. 3b). The WCB anomaly of the EMF (EHF) reaches on the order of 40% (50%) of the climatological maximum in the upper troposphere, as inferred from the 95th-percentile (5.20 day^−1^) and the anomaly color-bar range. This suggests that the eddy-driven modulation represents a sizable fraction of the mean activity in both the zonal-mean and horizontal patterns.

These changes are consistent with shifts in the WCB inflow, ascent, and outflow regions relative to the climatological structure (Supplementary Fig. 1). The inflow anomaly is relatively stronger on the upstream side of the climatological WCB, particularly over the North Pacific, whereas the outflow anomaly appears more clearly in the downstream region. These features suggest that under enhanced EMF conditions, WCBs tend to travel farther poleward and eastward before undergoing their main ascent. In fact, the ascent rate of WCB trajectories decreases by approximately 0.08 Pa h^−1^ km^−1^ per unit increase in EMF over the subtropics (25°N–50°N), while it increases by about 0.15 Pa h^−1^ km^−1^ in the subpolar region (50°N–70°N; Supplementary Fig. 3). Here, the ascent rate is defined as the pressure change per hour per unit horizontal distance traveled, computed along each trajectory at 6-hourly intervals. Physically, the ascent rate indicates how steeply WCB trajectories ascend per unit horizontal distance. We find that the spatial pattern of the mean WCB trajectory ascent rate is consistent with the EMF-related changes in the ascent rate of the rising branch of the TEM streamfunction (Fig. 8a in Yoo and Lee^9^), featuring a dipole of weakened ascent rate in the midlatitudes and strengthened ascent rate in the subpolar region.

When regressed onto the EHF index, the zonal-mean WCB frequency exhibits a vertical dipole structure, with positive anomalies above approximately 800 hPa and negative anomalies in the low-level layer below 800 hPa (Fig. 3c). However, unlike the EMF-related WCB anomalies, the horizontal separation between inflow, ascent, and outflow is less distinct in the EHF-related anomalies of the WCB (Fig. 3d and Supplementary Fig. 2). This structure contrasts with the more vertically differentiated (i.e., less barotropic) WCB anomalies associated with the EMF.

Because the near-surface anomaly corresponds to the inflow and the upper-level anomaly to ascent and outflow, the vertical dipole in Fig. 3c indicates an upward shift in the vertical distribution of WCB frequency under enhanced EHF conditions. Since WCBs originate near the surface, this redistribution reflects a more rapid ascent through the lower troposphere, resulting in reduced WCB residence at low levels and enhanced outflow frequency aloft. Consistent with this interpretation, enhanced mean ascent is observed in the regions of increased outflow frequency (Supplementary Figs. 2 and 3c). However, the zonal-mean WCB ascent rate anomaly exhibits weak negative values owing to strong zonal asymmetry (Supplementary Fig. 3). Meridionally, the zonal-mean WCB frequency anomalies are centered between about 40°N and 50°N, near or slightly poleward of the climatological maximum, matching the TEM streamfunction associated with the EHF (Fig. 3c).

To explore possible physical links between the eddy fluxes and the WCB, we examine the spatial correspondence between WCB frequency and vertical velocity anomalies (Supplementary Fig. 4). The two fields exhibit remarkably similar patterns, with positive (negative) WCB anomalies collocated with regions of anomalous ascent (subsidence), for both the EMF and EHF indices (shading). This correspondence likely arises because, although the WCBs are identified from Lagrangian trajectories, their detection depends on the Eulerian vertical velocity that is sufficiently strong and spatially and temporally coherent to lift air parcels above the threshold. In the zonal-mean sense, however, variations in WCB frequency correspond more closely to the TEM vertical velocity rather than to the Eulerian-mean velocity. This is because the TEM formulation explicitly incorporates the effect of eddies, including synoptic-scale cyclones within which WCBs are embedded.

We note that the WCB frequency anomalies appear to be associated with planetary-scale stationary waves (Supplementary Figs. 5–6). This association arises because, in our December–February (DJF) decomposition of the eddy fluxes (Eqs. 5–6), the stationary-eddy component exhibits the largest partial regression slope, accounting for approximately 62% (78%) of the total magnitude of the fitted EMF (EHF) indices. In contrast, the transient-eddy component contributes only about 14% (6%), while the covariance terms contribute to 24% (16%) to the fitted EMF (EHF) indices, respectively. As a result, when the regression analysis is performed using the transient flux component, the amplitudes of the WCB frequency anomalies are less than half of those obtained using the stationary component. This does not imply that WCBs themselves are stationary. Rather, it suggests that WCB occurrence and its associated flux anomalies can be modulated by large-scale stationary eddies. For example, WCB activity can be influenced by the Madden–Julian Oscillation through its impact on the larger-scale flow configuration^14^. Further analysis is required to fully elucidate the mechanisms underlying this behavior.

### Role of WCB in precipitation change

**Fig. 4:**
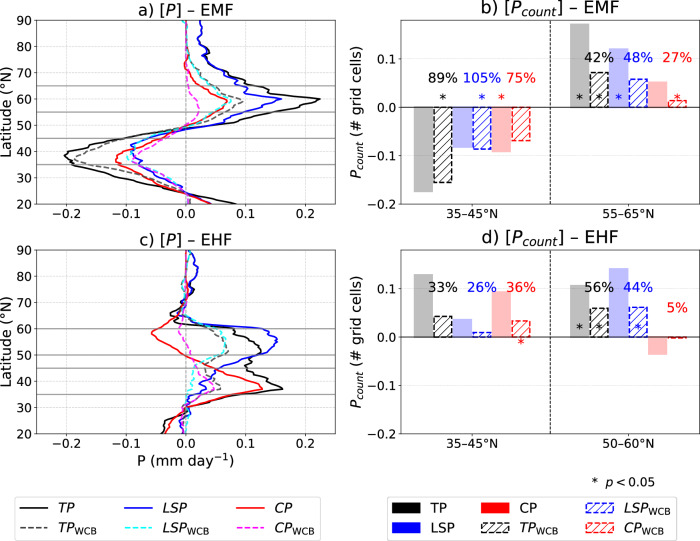
Regression of zonal-mean precipitation onto EMF and EHF. Regression of daily zonal-mean precipitation and precipitation frequency bins onto the EMF (top panels) and EHF (bottom panels) indices. **a**, **c** Regression is performed for total precipitation (TP; black), large-scale precipitation (LSP; blue), and convective precipitation (CP; red), along with their WCB-related counterparts (TP_WCB_, gray dashed; LSP_WCB_, cyan dashed; CP_WCB_, magenta dashed). **b**, **d** Regression of daily P_count_ (TP, LSP, and CP) onto b) EMF and d) EHF, integrated over 35–45°N and 55–65°N for EMF and 35–45°N and 50–60°N for EHF. Hatched bars show the corresponding WCB-related components (TP_WCB_, LSP_WCB_, CP_WCB_). Percent values above each bar indicate the fractional contribution of TP_WCB_ to TP, LSP_WCB_ to LSP, and CP_WCB_ to CP within each latitude band.

Previous studies have shown that the eddy-flux-driven TEM vertical motion plays an important role in shaping the meridional distribution of zonal-mean precipitation^9,10^. The EMF, in particular, exerts a stronger influence on precipitation than the EHF because the large-scale and convective components vary in phase and therefore add constructively, resulting in a stronger total precipitation anomaly (solid lines in Fig. 4a, c). In contrast, for the EHF, these components tend to offset one another, especially over 50°N–70°N. Regarding WCB, the slantwise ascending airstreams produce large-scale stratiform precipitation while also containing embedded convective bursts^8,19^. This physical linkage suggests that the in-phase increase of large-scale and convective precipitation under EMF forcing arises primarily from changes in WCB activity, whereas this in-phase behavior is largely absent under enhanced EHF forcing. Here, we quantify the WCB-related precipitation changes associated with the eddy fluxes.

WCB activity accounts for a substantial portion of the EMF-related precipitation changes, explaining 89% and 42% of the total anomalies within the latitudinal bands of 35°N–45°N and 55°N–65°N, respectively (Fig. 4a, b; see also Supplementary Fig. 7 for its spatial pattern). In the 35°N–45°N band, WCB-related zonal mean precipitation is nearly equally divided between large-scale and convective components, although the large-scale contribution is more pronounced in its spatial structure (Supplementary Fig. 8). In the 55°N–65°N band, the convective component contributes roughly one-third of the large-scale WCB-related precipitation, but both components again share the same sign.

For the EHF, WCB activity explains 33% and 56% of the precipitation changes within the latitudinal bands of 35°N–45°N and 50°N–60°N, respectively (Fig. 4c, d). In the 35°N–45°N band, both the large-scale and convective components show positive anomalies. In contrast, in the 50°N–60°N band, the large-scale precipitation increases in response to the enhanced EHF, whereas the convective component is nearly zero or slightly negative.

We speculate that the contrasting response between the large-scale and convective precipitation components is linked to changes in the ascent rate of WCB trajectories and their interaction within the surrounding thermodynamic environment of the cyclone system. Steeper, more upright WCB trajectories (discussion in Section 3.2) confine the ascent to a shorter horizontal distance, thereby promoting rapid saturation and enhanced large-scale precipitation near the ascent region (Supplementary Fig. 8). At the same time, the reduced residence time in the lower troposphere, evidenced by vertical dipole structure (Fig. 3c), may limit the moisture uptake from the boundary layer, while earlier and higher-level detrainment decreases the availability of moist, conditionally unstable air that could support downstream convective development^20^. As a result, unlike the large-scale precipitation, the convective component shows a weak response in the far downstream region (Supplementary Fig. 8). We note that this speculation does not consider other factors that influence boundary-layer moisture supply, such as sea surface temperature and the characteristics of airstreams feeding the WCB inflow region.

## Discussion

This study demonstrates a close dynamical coupling between synoptic-scale WCB activity and the large-scale TEM overturning circulation in Northern Hemisphere winter. In particular, WCB frequency covaries with TEM ascent and exhibits distinct responses under EMF and EHF forcing. Enhanced EMF is associated with a meridional displacement of preferred WCB ascent, whereas enhanced EHF primarily strengthens WCB activity downstream over the Pacific with comparatively weak meridional displacement. This EHF response may reflect zonal asymmetries in stationary-eddy heat transport over the Pacific^21^, which modulate storm-track intensity and WCB occurrence more strongly in longitude than in latitude. In both cases, the responses are strongly shaped by planetary-scale stationary waves.

WCBs also play a central role in mediating the precipitation responses associated with the TEM circulation. WCB-related precipitation accounts for 89% of the EMF-induced precipitation anomalies in the 35°N–45°N band and 42% in the 55°N–65°N band. WCBs likewise contribute substantially to the EHF-induced precipitation anomalies (33% in 35°N–45°N and 56% in 50°N–60°N), although in the 50°N–60°N band the convective component of WCB-related precipitation is small. How WCB-related precipitation responds to large-scale eddy forcings, and hence the TEM–precipitation connection, likely depends on changes in the vertical distribution and ascent characteristics of WCB trajectories. For example, the precipitation associated with the WCB may be modulated by changes in ascent rate, moisture availability, or the spatial distribution of inflow and ascent. This topic warrants further investigation.

The results highlight a connection between synoptic-scale WCBs and the large-scale TEM circulation. The two can be viewed as complementary expressions of the same underlying dynamics. WCBs describe the three-dimensional ascent of moist air, while the TEM circulation represents the residual overturning that emerges from eddy forcings and diabatic heating. Thus, the TEM offers a useful way to interpret the statistics of WCB activity in both the climatological mean and in response to changes in eddy fluxes. At the same time, the TEM description is inherently zonal-mean and cannot capture the full longitudinal structure, intermittency, or lifecycle evolution of individual WCB events.

Recently, the first climatologies of WCBs in climate models have been explicitly computed, using both direct trajectory calculations^18^ and alternative detection approaches based on machine learning^22^. While these methods provide valuable and increasingly accessible tools for diagnosing WCB behavior, they can be computationally demanding or methodologically complex when applied across large ensembles or many climate-model scenarios. In this context, a TEM-based perspective offers a complementary and physically grounded framework for interpreting WCB-related changes in the zonal-mean circulation and precipitation, and a practical baseline diagnostic when explicit WCB tracking is infeasible.

In a warmer, moister climate, WCBs are expected to become more precipitation efficient because increased water vapor promotes earlier saturation and larger latent heat release during ascent. Consequently, WCB-related precipitation, including its contribution to extremes, can intensify even if WCB frequency changes only modestly. This thermodynamic amplification may also project onto the large-scale circulation, since stronger diabatic heating in storms can strengthen the effective rising branch of the TEM overturning and shift it as storm tracks and jets migrate through WCB-related diabatic PV generation^18,23^. Accordingly, we expect warming to strengthen the relationship between WCB-related precipitation and TEM ascent, whereas the relationship between WCB frequency and TEM ascent may change less. However, warming can also induce dynamical reorganization. In latitudes where TEM ascent weakens due to changes in eddy forcing even as background moisture increases, WCB precipitation may still increase without a proportional intensification of TEM ascent. Diagnosing whether models reproduce these coupled WCB–TEM responses can provide a process-oriented evaluation of storm-track and jet projections and their associated precipitation changes.

## Methods

### Reanalysis data

We employ the fifth generation of the atmospheric reanalysis product from the European Centre for Medium-Range Weather Forecasts^24^ (ERA5) for December–February (DJF) during 1980/81 to 2021/22. Winter is chosen because midlatitude eddy activity and WCB occurrence are the strongest during this season. The data are originally provided at a horizontal resolution of 0.25° × 0.25°, with six-hourly temporal intervals and 37 vertical pressure levels (1–1000 hPa). They are then regridded to 1.5° × 1.5° and converted to daily means. The analyzed variables are three-dimensional wind components, temperature, and precipitation.

### WCB trajectories

We use the WCB trajectory dataset^19,25^, generated with the LAGRANTO trajectory tool^26^ using ERA5 reanalysis winds. The dataset is publicly available^27^. In this dataset, kinematic trajectories were initiated every six hours and integrated forward in time for 96 h using the three-dimensional ERA5 winds with hourly temporal resolution, 0.5° horizontal resolution, and 137 model levels extending from the surface to 0.1 hPa. At each initialization time, trajectories were launched globally from the lower troposphere (1050–790 hPa) with a horizontal spacing of approximately 80 km and a vertical spacing of 20 hPa. Trajectories that ascend by at least 600 hPa within 48 h are identified as WCBs. Additional filters remove duplicate trajectories and ensure that the ascent occurs within the vicinity of extratropical cyclones^7,19^.

For the present analysis, the original six-hourly WCB trajectories were converted into gridded WCB frequency fields (0.25° × 0.25°) following the WCB mask methodology^19^. Specifically, we compute the fraction of grid points experiencing WCB inflow, ascent, and outflow phases, defined here by pressure: inflow (below 800 hPa), ascent (between 800 and 400 hPa), and outflow (above 400 hPa). These frequency fields were then interpolated onto a 1.5° × 1.5° grid and averaged into daily means. Note that atmospheric rivers (ARs), which are typically diagnosed as Eulerian filaments of anomalously strong vertically integrated vapor transport, often coincide with the low-level moist inflow that can feed WCB ascent. However, the relationship is not one-to-one: ARs can occur without a clearly identified ascending WCB airstream, and WCB ascent and associated precipitation do not necessarily align with the AR axis^7,28,29^.

### TEM streamfunction and eddy fluxes

The TEM streamfunction ($\left[{\psi }^{r}\right])$ is obtained by vertically integrating the residual meridional wind ($\left[{v}^{r}\right]$) downward from the top of the atmosphere:

1 $$\left[{\psi }^{r}\right]\left(t,\phi \right)=\frac{2\pi a\,\cos \phi }{g}{\int }_{0}^{p}\left[{v}^{r}\right]\left(t,p,\phi \right){dp}$$

where *a* is the Earth’s radius, *g* is the gravitational acceleration, *ϕ* is latitude, *p* is pressure, and *t* is time. Here, the bracket denotes the zonal mean, and superscript *r* indicates the residual component. The residual meridional wind is defined as

$$\left[{v}^{r}\right]=\left[v\right]-{\left(\left[{v}^{* }{\theta }^{* }\right]/{\left[\theta \right]}_{p}\right)}_{p}$$

where *θ* indicates the potential temperature and the asterisk the deviation from the zonal mean. By the zonal-mean continuity equation, the residual vertical wind can be defined from the streamfunction as,

2 $$\left[{{\rm{\omega }}}^{r}\right]=\frac{g}{2\pi {a}^{2}\cos \phi }\frac{\partial }{\partial \phi }\left(\left[{\psi }^{r}\right]\cos \phi \right)$$

Because the TEM streamfunction in the midlatitude is strongly influenced by the meridional eddy fluxes of momentum and heat, we construct indices representing the strength of poleward eddy momentum and heat fluxes^9^. The momentum and heat indices are defined respectively as

3 $${I}_{M}\left(t\right)={\sum }_{\phi ,p}{\left[{u}^{* }{v}^{* }\right]}_{\mathrm{DJF}}\left[{u}^{* }{v}^{* }\right]\left(t,\phi ,p\right)\cos \phi$$

and

4 $${I}_{H}\left(t\right)={\sum }_{\phi ,p}{\left[{v}^{* }{\theta }^{* }\right]}_{\mathrm{DJF}}\left[{v}^{* }{\theta }^{* }\right]\left(t,\phi ,p\right)\cos \phi$$

Here, the subscript DJF indicates the DJF climatology.

The eddy field can be decomposed further into its stationary $\overline{\left(\cdot \right)}$ and transient ${\left(\cdot \right)}^{{\prime} }$ components as

$${v}^{* }=\bar{{v}^{* }}+{v}^{{\prime} }$$

Then, the eddy fluxes in Eqs. (3–4) can be written as the sum of the stationary flux, the transient flux, and the cross terms:

5 $$\left[{u}^{* }{v}^{* }\right]=\left[\bar{{u}^{* }}\bar{{v}^{* }}\right]+\left[{u}^{{\prime} }{v}^{{\prime} }\right]+\left[\bar{{u}^{* }}{v}^{{\prime} }\right]+\left[{u}^{{\prime} }\bar{{v}^{* }}\right]$$

and

6 $$\left[{v}^{* }{\theta }^{* }\right]=\left[\bar{{v}^{* }}\bar{{\theta }^{* }}\right]+\left[{v}^{{\prime} }{\theta }^{{\prime} }\right]+\left[{v}^{{\prime} }\bar{{\theta }^{* }}\right]+\left[\bar{{v}^{* }}{\theta }^{{\prime} }\right]$$

The stationary component is extracted using a 30-day Lanczos low-pass filter with 90-day weighting window.

### WCB-related precipitation and binning

We define precipitation occurring within a certain radius of 48-h WCB trajectories as being associated with the WCB. A typical threshold value adopted in previous studies is 100 km, reflecting the characteristic horizontal scale of the WCB^7,12,18,30^. In this study, given our horizontal resolution of 1.5°, we include the four surrounding grid points around each WCB trajectory when applying the WCB mask to precipitation (P_WCB_).

To quantify how P_WCB_ contributes to the precipitation (P) response in latitude bands, daily precipitation at each grid point is first converted into a time series of counts using the precipitation-bin framework. For each latitude band, the counts from all grid points within the band are summed over longitudes to produce band-integrated time series for both P and P_WCB_. These latitude-band count time series are then regressed onto the daily EMF and EHF indices to assess how precipitation occurrence within each band varies with changes in eddy forcing. The contribution of P_WCB_ to P is obtained by comparing the P_WCB_ regression with the corresponding P regression for the same latitude band.

## Supplementary information

Supplementary Information.

## Data Availability

The daily gridded WCB frequency fields used in this study are archived on Zenodo (10.5281/zenodo.18752738). ERA5 data are available from the Copernicus Climate Data Store (https://cds.climate.copernicus.eu/).

## Code availability

WCB calculations were performed using the LAGRANTO software (https://iacweb.ethz.ch/staff/sprenger/lagranto/). The codes to reproduce the analyses presented in this study are available upon request from the corresponding author.
