# Supplementary material for: Linking warm conveyor belts to the TEM circulation in Northern Hemisphere midlatitude winter
Source: NPJ Clim Atmos Sci. 2026 May 15;9(1):164. doi: 10.1038/s41612-026-01435-3 (PMC13433280; doi:10.1038/s41612-026-01435-3)
Supplement: Supplementary file 1 [file 41612_2026_1435_MOESM1_ESM.pdf]

Supplementary Information for

# Linking Warm Conveyor Belts to the TEM Circulation in Northern Hemisphere Midlatitude Winter

Yunsung Hwang[1], Changhyun Yoo[1,2*], Sukyoung Lee[3], Michael Sprenger[4], and Marc Federer[5]

[1] *Severe Storm Research Center, Ewha Womans University, Seoul, South Korea*

[2] *Dept. of Climate and Energy Systems Engineering, Ewha Womans University, Seoul, Republic of Korea*

[3] *Dept. of Meteorology and Atmospheric Science, The Pennsylvania State University, University Park, Pennsylvania, USA*

[4] *Institute for Atmospheric and Climate Science, ETH Zurich, Switzerland*

[5] *Dept. of Earth, Atmospheric and Planetary Sciences, Massachusetts Institute of Technology, Cambridge, Massachusetts, USA*

**Contents of this file**

Supplementary Figures 1–8

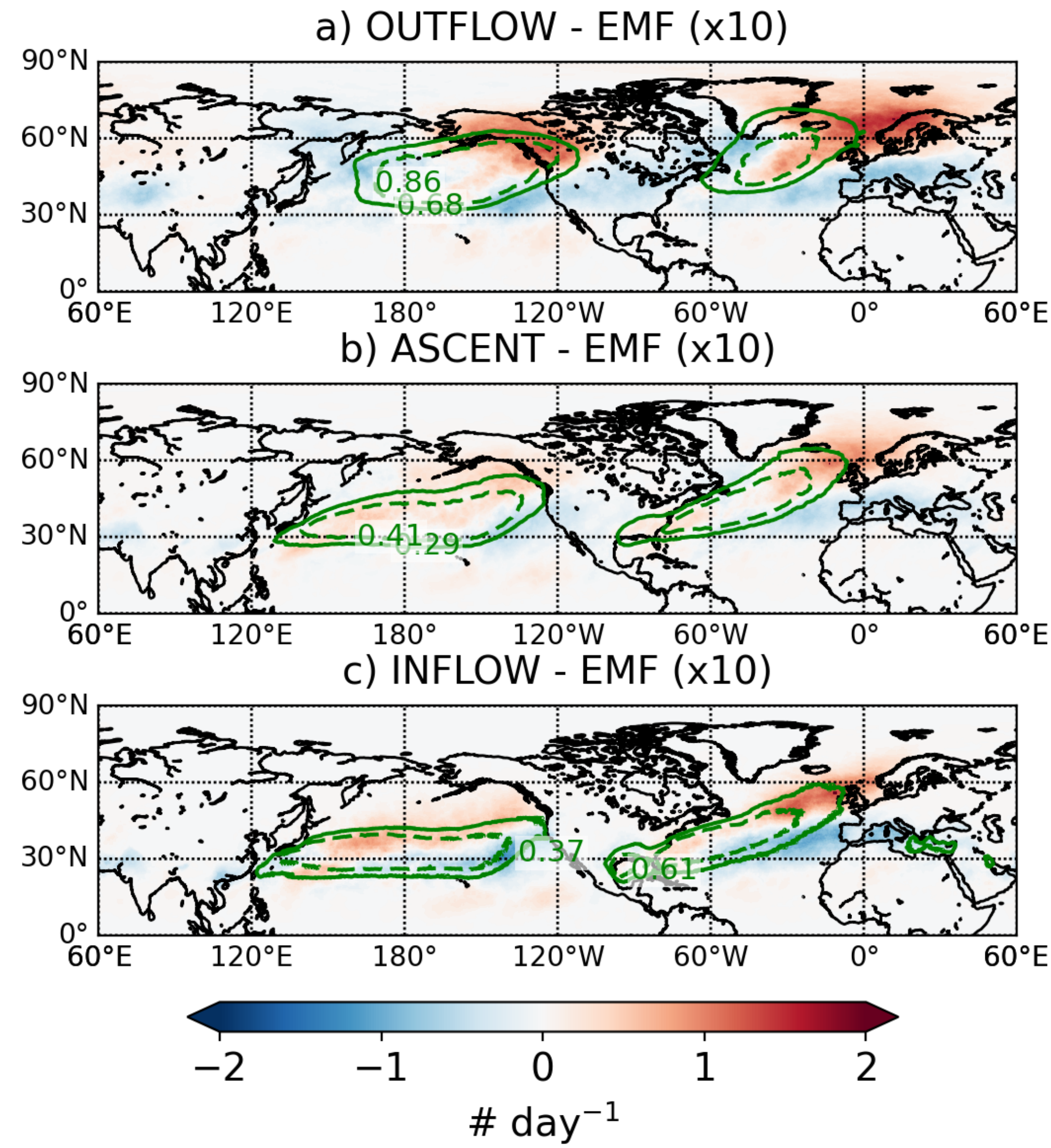


**Supplementary Figure 1.** As in Fig. 3b, but the regression is performed against the EMF for a) the WCB outflow, b) the WCB ascent, and c) the WCB inflow frequencies, respectively. Green contours show the climatological WCB frequency, with 90$^{th}$ (solid) and 95$^{th}$ (dashed) percentile values of 0.68 and 0.86 day$^{-1}$ for outflow, 0.29 and 0.41 day$^{-1}$ for ascent, and 0.37 and 0.61 day$^{-1}$ for inflow, respectively.

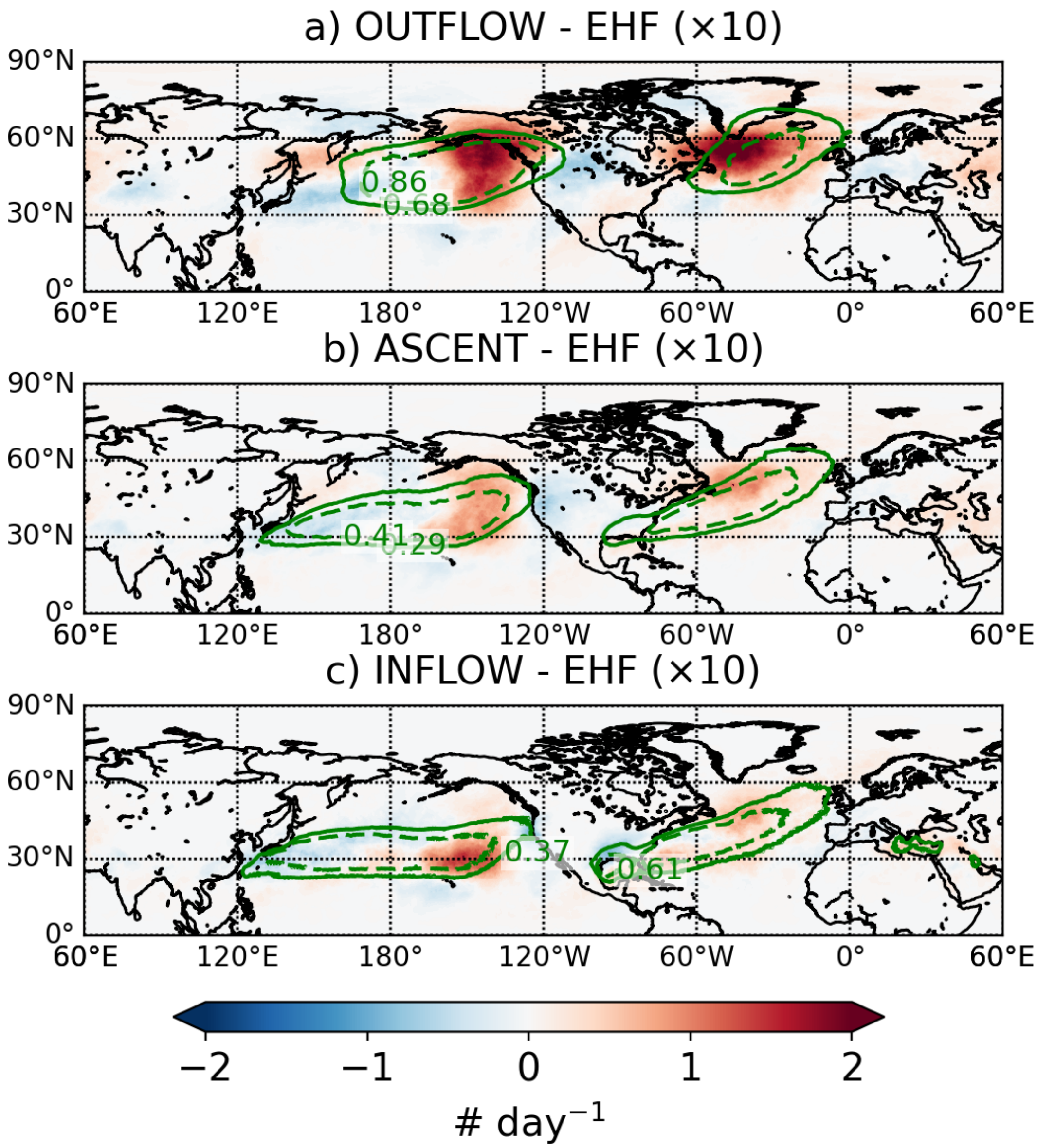


**Supplementary Figure 2.** As in Fig. 3d, but the regression is performed against the EHF for a) the WCB outflow, b) the WCB ascent, and c) the WCB inflow frequencies, respectively.

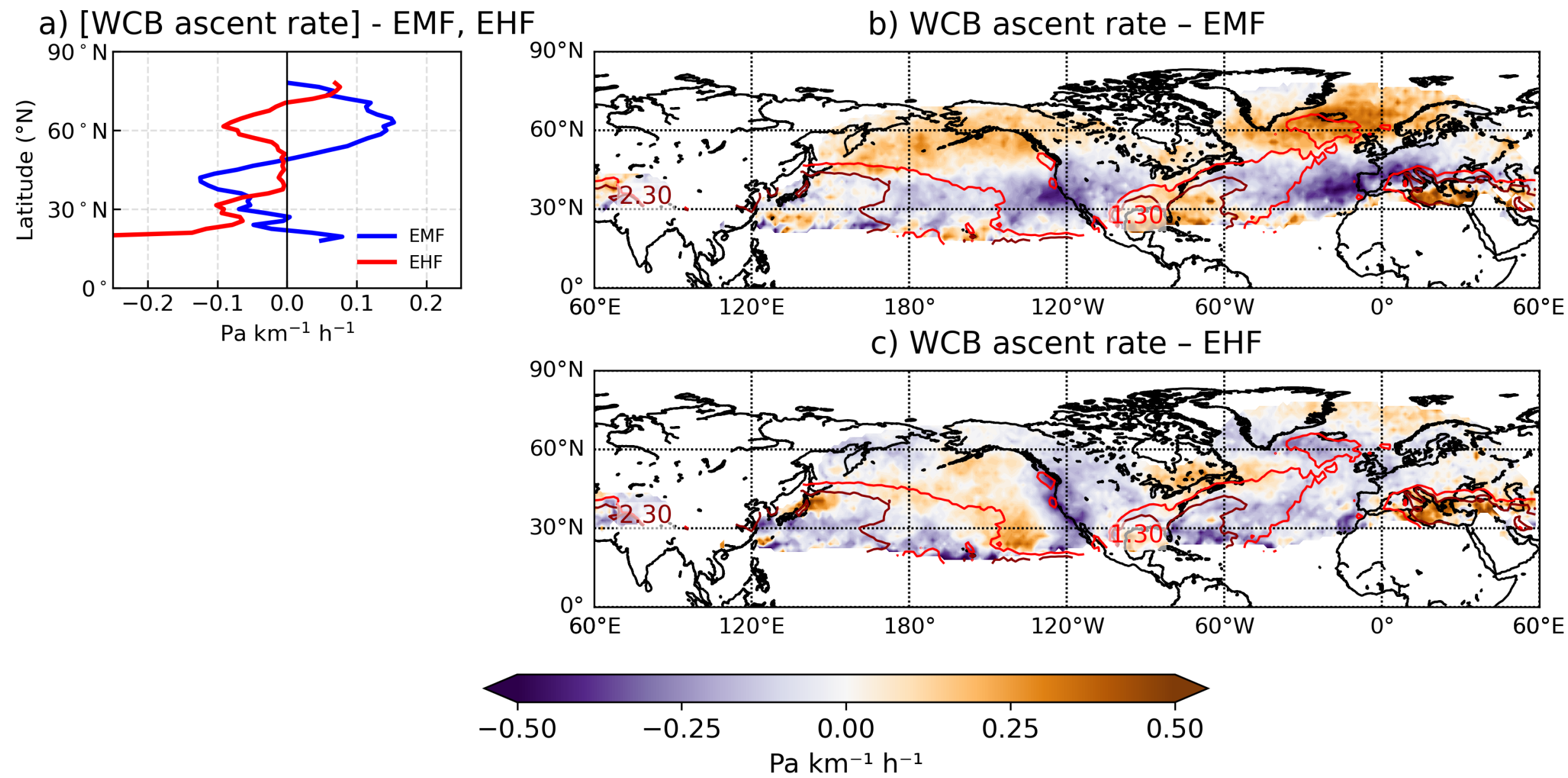


**Supplementary Figure 3.** WCB ascent rates, defined as the pressure change per hour per unit horizontal distance traveled along WCB trajectories (Pa $km^{-1}$ $h^{-1}$), regressed onto EMF and EHF indices. Panel a) shows the zonal-mean sensitivity of the WCB ascent rate to EMF (blue) and EHF (red). Panels b)–c) present the spatial regression patterns of the WCB ascent rate onto EMF and EHF, respectively. Red and dark-red contours denote the climatological WCB ascent rate values of 1.0 and 2.0 Pa $km^{-1}$ $h^{-1}$, respectively.

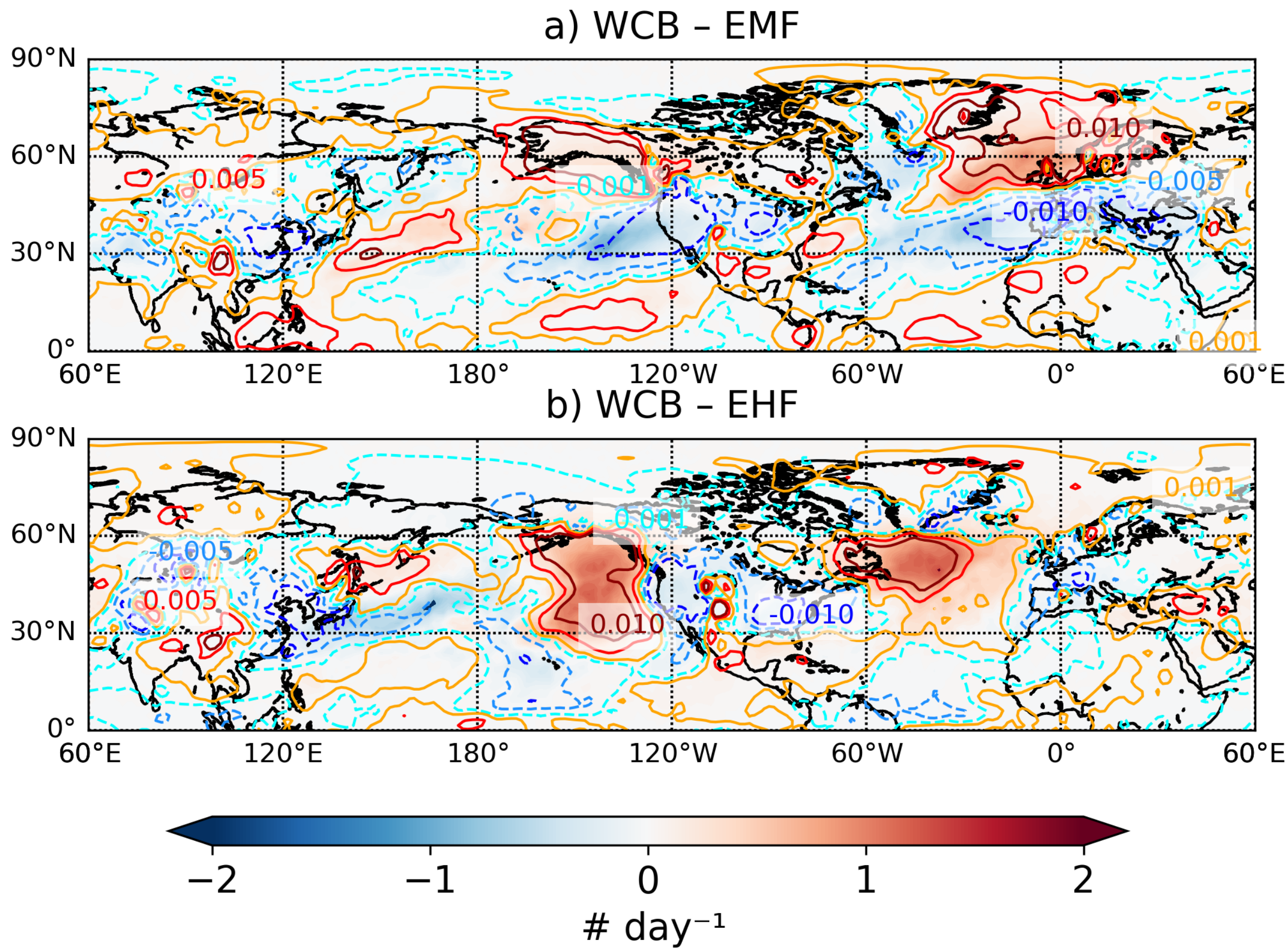


**Supplementary Figure 4.** Regression of WCB frequency onto a) EMF and b) EHF during DJF. Colored contours show ($-\omega_{500}$) regressed onto EMF and EHF. Negative regression values are shown in blue, dodgerblue, and cyan, corresponding to −0.01, −0.005, and −0.001 Pa s$^{-1}$, while positive values are shown in orange, red, and darkred, corresponding to 0.001, 0.006, and 0.01 Pa s$^{-1}$.

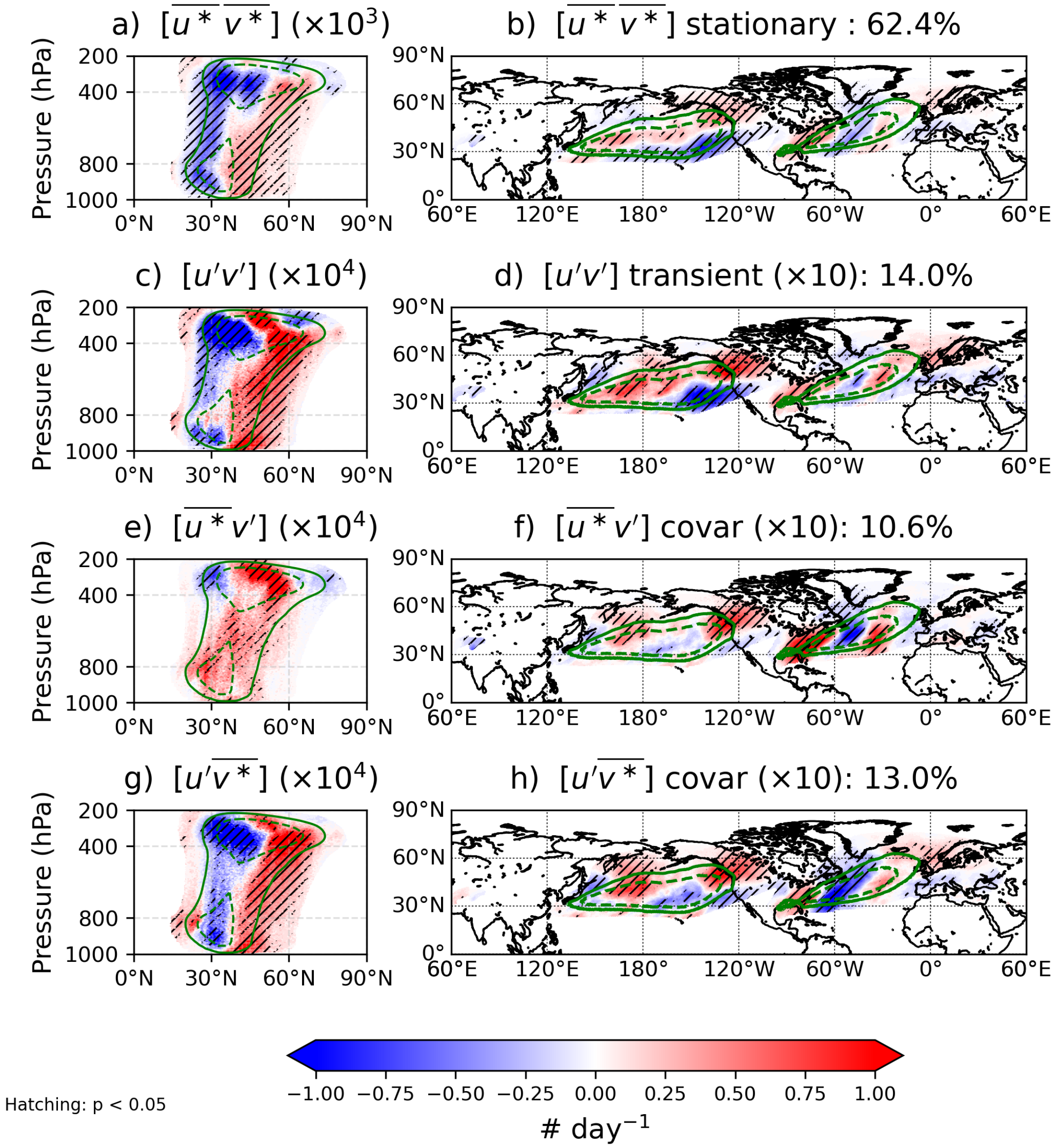


**Supplementary Figure 5.** Regression of WCB frequency onto the decomposed EMF components during DJF. Shading shows regression slopes (# day$^{-1}$) for a) and b) stationary $[\overline{u^*}\,\overline{v^*}]$, c) and d) transient $[u'v']$, e) to h) covariance $[u'\overline{v^*}]$ and $[\overline{u^*}\,v']$ EMF indices. Green contours outline the 90$^{th}$ (solid) and 95$^{th}$ (dashed) percentiles (0.006 and 0.02 day$^{-1}$ for the first column and 3.36 and 5.20 day$^{-1}$ for the second column) of the climatological WCB frequency. Percentages denote the relative regression weights of each EMF component in a multiple linear regression onto the total EMF index. Hatching marks regions where the WCB–EMF regression is significant at the 95% confidence level.

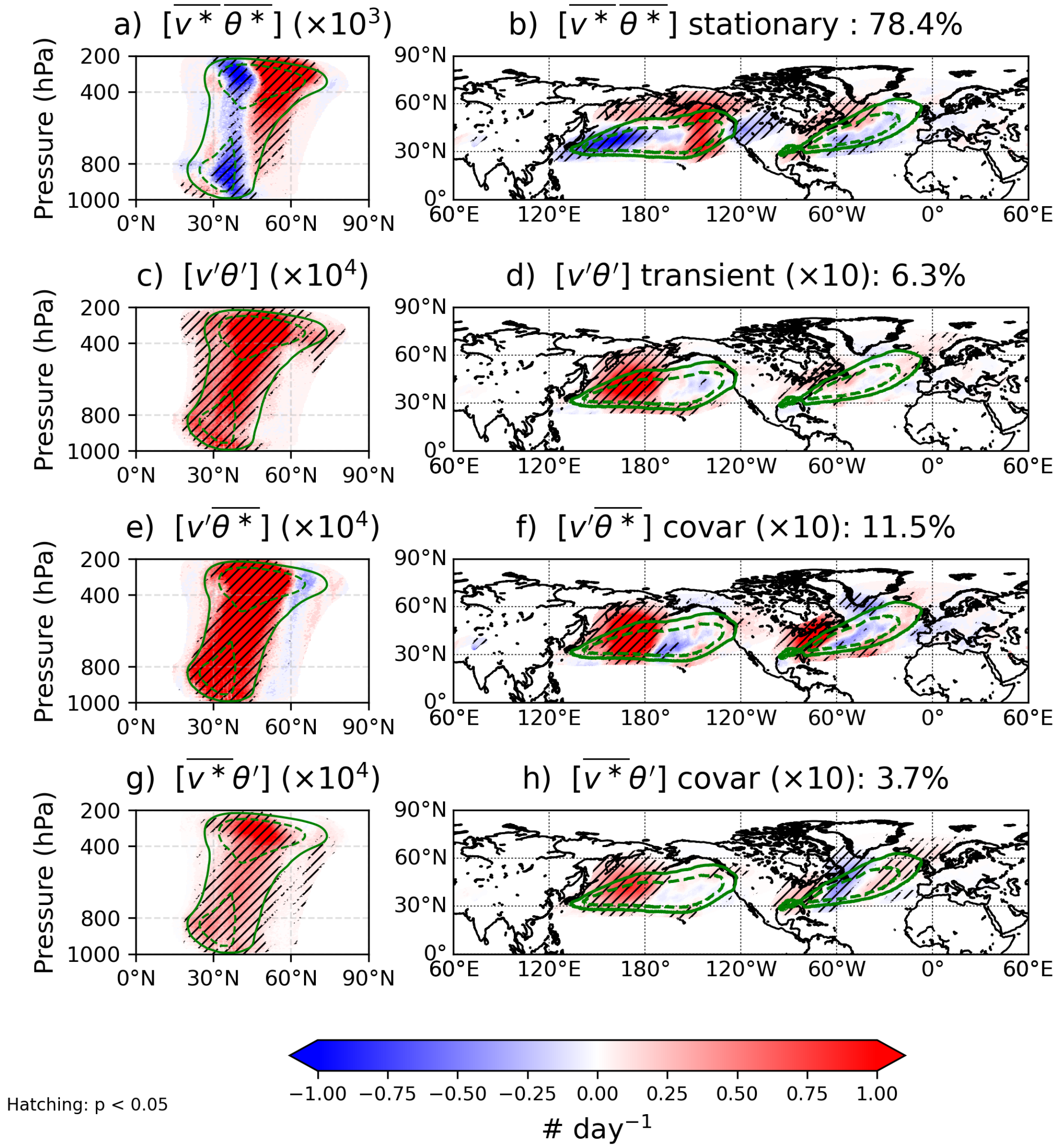


**Supplementary Figure 6.** Same as Supplementary Fig. 5, but for the decomposition of EHF.

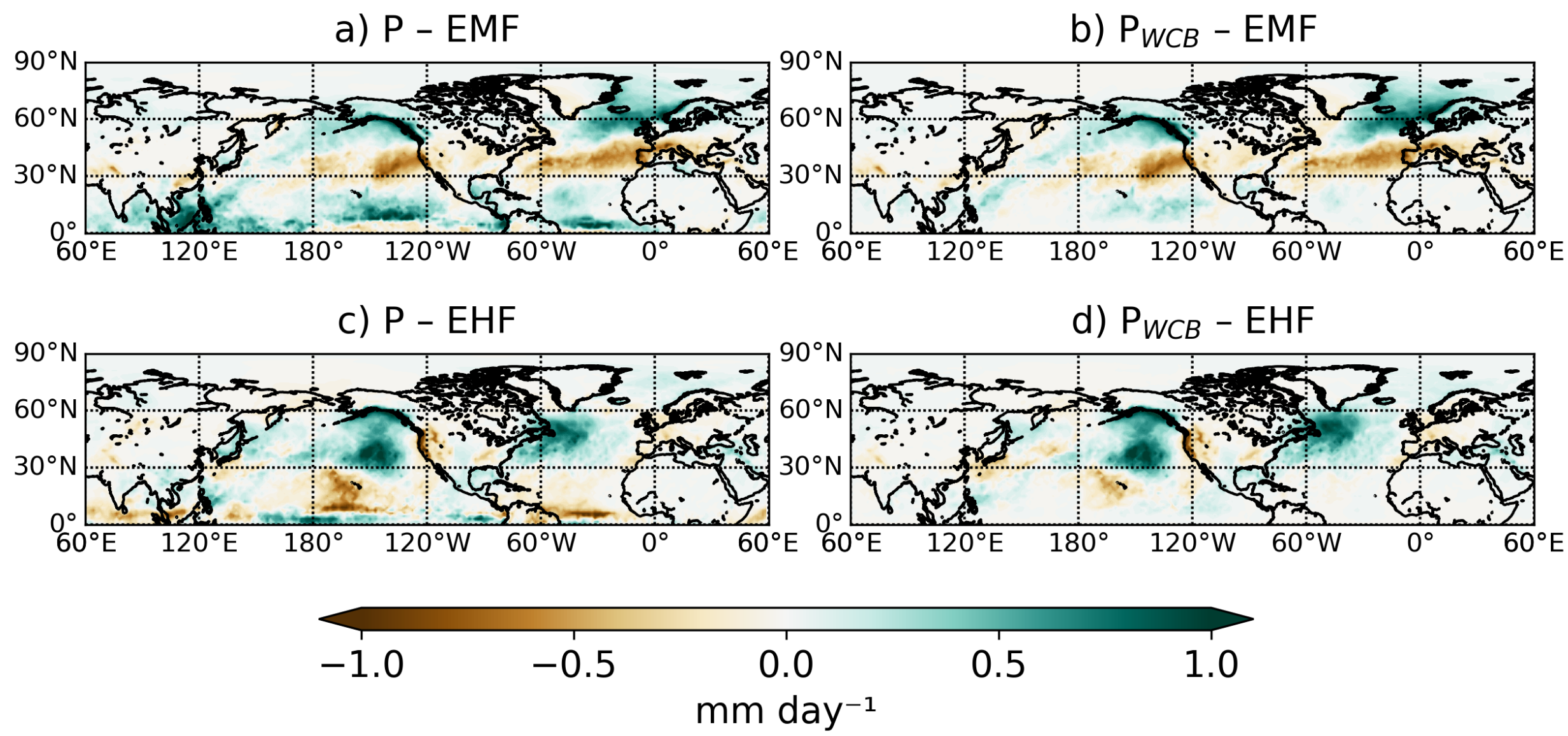


**Supplementary Figure 7.** Regression of TP and $TP_{WCB}$ onto EMF and EHF indices during DJF (1980–2022). Panels a)–b) show the regression slopes of TP and $TP_{WCB}$ with respect to EMF, while panels c)–d) show the corresponding slopes with respect to EHF.

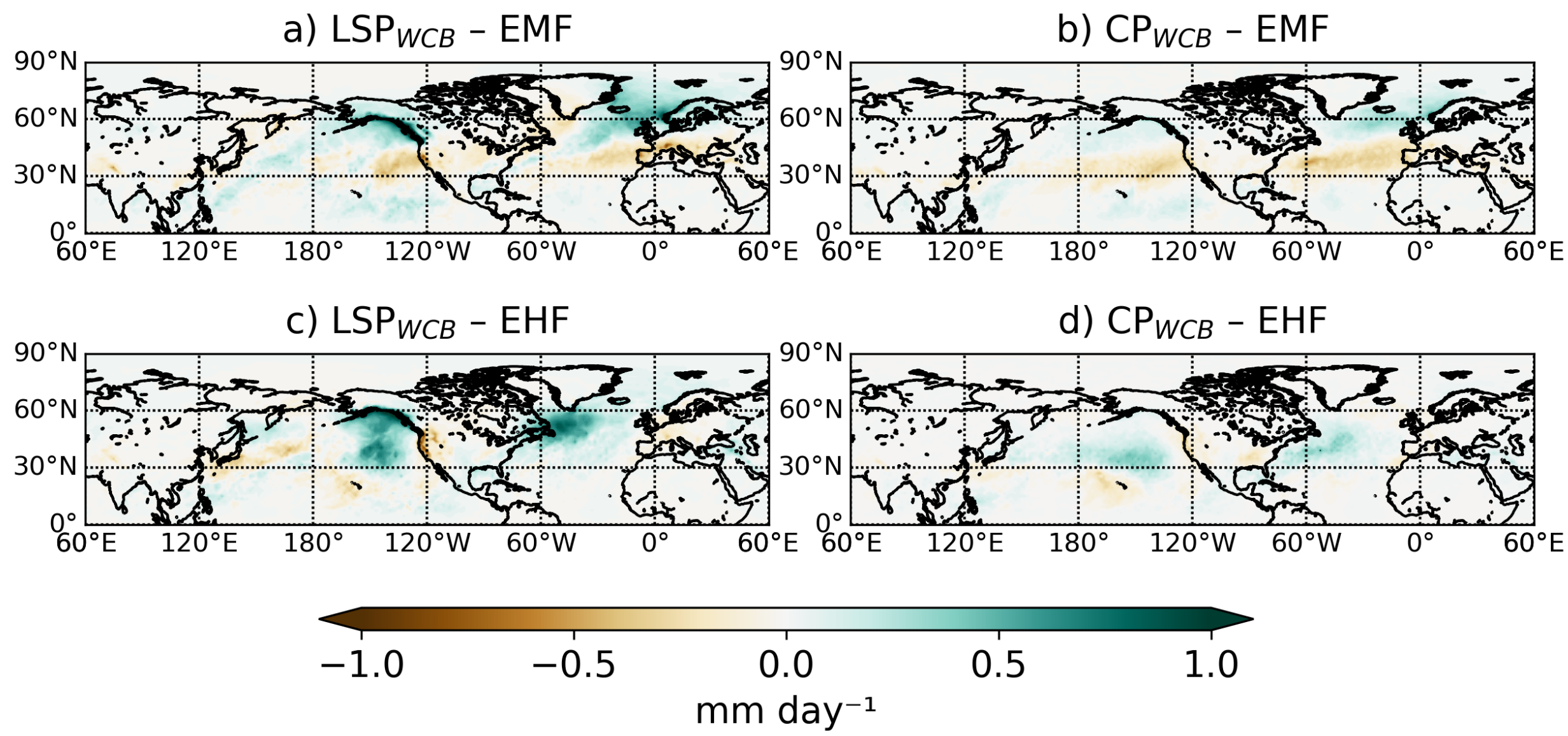


**Supplementary Figure 8.** Same as Supplementary Fig. 7, but showing a) $LSP_{WCB}$ regressed to EMF, b) $CP_{WCB}$ regressed to EMF, c) $LSP_{WCB}$ regressed to EHF, and d) $CP_{WCB}$ regressed to EHF.
